# Critical Issues of Double-Metal Layer Coating on FBG for Applications at High Temperatures

**DOI:** 10.3390/s19183824

**Published:** 2019-09-04

**Authors:** Carla Lupi, Ferdinando Felli, Alessandro Dell’Era, Erwin Ciro, Michele Arturo Caponero, Hypolito José Kalinowski, Cristian Vendittozzi

**Affiliations:** 1 Department ICMA, Sapienza University of Rome, 00184 Rome, Italy; 2 Department SBAI, Sapienza University of Rome, 00161 Rome, Italy; 3 ENEA CR Frascati, Frascati, 00044 Rome, Italy; 4 Universidade Federal Fluminense, Rio de Jaineiro, 24210-240 Niterói, Brazil; 5 Universidade de Brasília, 72444-240 Gama Brasília-DF, Brazil; vendittozzi@aerospace.unb.br

**Keywords:** high temperature FBG sensors, electrodeposition, metal coating, harsh environment Sensors

## Abstract

Use of fiber Bragg gratings (FBGs) to monitor high temperature (HT) applications is of great interest to the research community. Standard commercial FBGs can operate up to 600 ${}^{\circ }$C. For applications beyond that value, specific processing of the FBGs must be adopted to allow the grating not to deteriorate. The most common technique used to process FBGs for HT applications is the regeneration procedure (RP), which typically extends their use up to 1000 ${}^{\circ }$C. RP involves a long-term annealing of the FBGs, to be done at a temperature ranging from 550 to 950 ${}^{\circ }$C. As at that temperature, the original coating of the FBGs would burn out, they shall stay uncoated, and their brittleness is a serious concern to deal with. Depositing a metal coating on the FBGs prior to process them for RP offers an effective solution to provide them with the necessary mechanical strengthening. In this paper, a procedure to provide the FBG with a bimetallic coating made by copper and nickel electrodeposition (ED) is proposed, discussing issues related to the coating morphology, adherence to the fiber, and effects on the grating spectral response. To define the processing parameters of the proposed procedure, production tests were performed on dummy samples which were used for destructive SEM–EDS analysis. As a critical step, the proposed procedure was shown to necessitate a heat treatment after the nickel ED, to remove the absorbed hydrogen. The spectral response of the FBG samples was monitored along the various steps of the proposed procedure and, as a final proof test for adherence stability of the bimetallic coating, along a heating/cooling cycle from room temperature to 1010 ${}^{\circ }$C. The results suggest that, given the emergence of Kirkendall voids at the copper–nickel interface, occurring at the highest temperatures (700–1010 ${}^{\circ }$C), the bimetallic layer could be employed as FBG coating up to 700 ${}^{\circ }$C.

## 1. Introduction

Fiber Bragg gratings (FBGs) are fiber optic sensors whose operating principle is based on the Bragg diffraction effect [1]. Along a short segment (typical 10 mm) of the optical fiber, a diffraction grating is *written* to produce a periodic modulation of the refraction index of the core of the fiber itself. If broadband light propagates along the fiber, some narrow-band light is diffracted at the grating; the diffracted light counter-propagates along the fiber, as a wavelength-selective back-reflection. Production of FBGs is done by various techniques, the most common for commercial production being the irradiation of the fiber segment by ultraviolet (UV) light with a periodically modulated intensity pattern. The UV light modifies the refraction index of the core of the fiber, somehow accordingly to its modulated intensity pattern, thus producing the diffraction grating. The diffraction grating is stable at room temperature (RT) but suffers instability at high temperature (HT). Typically, critical issues affect FBG features and integrity at temperatures higher than 600 ${}^{\circ }$C, such as the migration of the doping elements within the silica matrix of the optical fiber, which affects the refraction index of the fiber core and cladding, or the silica re-crystallization, which leads to modification of the optical fiber structure and optical properties, and finally, the alteration of the refraction index modulation by which the FBG itself is produced. As a result, FBGs are subjected to thermal instability with a fast decay of their features. Such decay is triggered with no recovery even by a temporary reaching of HT, although this could also occur with a long exposure at lower (than 600 ${}^{\circ }$C) temperature. It was observed that starting from 600 to 700 ${}^{\circ }$C, the features of FBG decay until complete *erasure* of the FBG [2,3,4,5,6].

To *write* FBGs for HT (600–1000 ${}^{\circ }$C) applications, special techniques shall be adopted, and optical fibers less sensitive to thermal effect shall be considered. The most common technique used to produce FBGs for HT application is the regeneration procedure (RP). After the FBG has been *written* into the fiber to produce a grating with proper features, it is later exposed to HT for long time. This causes a *rearrangement* of the previously written FBG, which leads to its *erasure* and the generation of a higher temperature-resistant FBG; therefore, these types of FBGs are named *regenerated* [7,8]. This long-lasting thermal process (named annealing) is part of the RP and is critical to the production of the regenerated FBG. An annealing temperature of about 950 ${}^{\circ }$C is necessary for most germanium-doped fibers, whereas an annealing temperature of about 550 ${}^{\circ }$C is necessary for most co-doped fibers (germanium and boron doping). According to this, the optical features of the regenerated FBG are affected by the RP parameters. When the FBG undergoes an RP, its Bragg wavelength usually shifts toward lower values; the amount of the shift is related to the annealing temperature, and the higher the temperature the larger the shift. In co-doped fibers, the wavelength shift is proportional to the annealing temperature [9]; in germanium-doped fibers, the wavelength shift is proportional to the square of the annealing temperature. The highest temperature at which regenerated FBGs can operate depends on various factors (as, for instance, the fiber-dopant type) but never exceeds the temperature of the RP, which cannot be higher than 1000 ${}^{\circ }$C to avoid unsafe glass softening. Deposition of metal coating is a procedure of effective advantage in the production of regenerated FBGs [10]. In fact, the original coating of the FBG (typical: acrylate, polyimide, ormocer) would burn during the RP and shall thus be stripped; as a consequence, the brittleness of the uncoated FBG becomes a serious concern to deal with.

Depositing a metal coating on the FBGs prior to processing them for annealing offers an effective solution to provide them with the necessary mechanical strengthening. Moreover, the metal coating is also effective in increasing the temperature sensitivity of the regenerated FBG, which is useful if its use is intended for HT sensing [11,12,13,14,15]. Following these considerations, we tried to identify the most suitable metal for FBG coating starting from the most common and easily electrodeposited metals with a high melting temperature, such as copper (Cu) and nickel (Ni). The main purpose of this investigation was to search for the best metallic coating that would allow using the FBG for HT. The double layer of Cu and Ni was selected trying to achieve cupronickel alloys by means of interdiffusion between the two layers. The cupronickel coating would allow greater resistance in particularly harsh conditions. Starting from a Cu–Ni double metal layer, the investigation must state how the metal coating mechanically and thermally responds to temperature variations and how the coated fiber has been protected, enhancing or decreasing gratings’ performances.

## 2. Materials and Methods

The test campaign was carried out with samples produced using FBGs written on germanium-doped single-mode optical fiber (9 microns core, 125 microns cladding, 250 microns acrylate coating). The length of the FBG is about 10 mm. To produce the samples, the fiber was stripped (acrylate coating removal) for about 50 mm centered on the FBG. The stripped segment was first gilded by sputtering, and then a Cu layer was electrodeposited. Some sample was further processed, and a Ni layer was electrodeposited. Thus, two sets of samples were produced: one set of FBG samples with a single metallic (Cu) protective layer and one set of FBG samples with a bimetallic (Cu–Ni) protective layer. The samples with a single metallic layer were used as a reference to interpret the behavior of the samples with the bimetallic layer, thus identifying the specific issues due to the deposition of the second layer. During the test campaign, FBGs were continuously monitored using an FBG interrogation system with spectra acquisition capability (HBM-Fibersensing, Moreira, Portugal, model FS22 SI, providing a resolution of <0.5 pm, as stated in the manufacturer data-sheet). In addition to the FBG samples, dummy samples (optical fiber with no FBG) were also produced for both sets. The dummy samples were used for the optimization of the electrodeposition (ED) process parameters and for the metallographic (destructive) analysis that allowed the characterization of the metallic deposit, before and after the thermal cycles.

### 2.1. Metal Coating Process

In recent years, low-melting-point processes such as electroplating, vacuum brazing, sputtering, evaporation (CVD and PVD), ultrasonic consolidation, and electroless plating have been used for FBGs recoating [16,17,18,19,20]. In the present work, coating was made by ED. The esults of preliminary ED tests carried out on dummy samples and the relative morphological analysis led to considering as adequate an ED time of 240 min for Cu and Ni. Thus, the ED time is 240 min for the Cu FBG samples and 240 + 240 for the Cu–Ni FBG samples (altogether 480 min).

**Figure 1:**
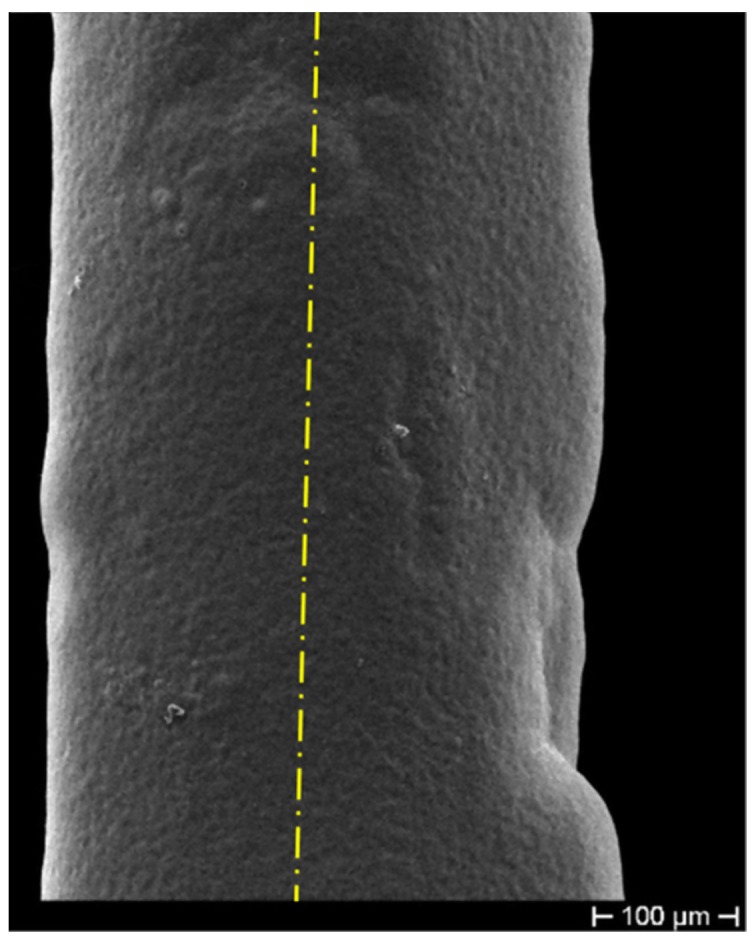
Non-uniform deposition due to a non-in-axis positioning of the cathode. The yellow dash-dotted line shows the actual position of the cylindrical Pb anode axis.

**Figure 2:**
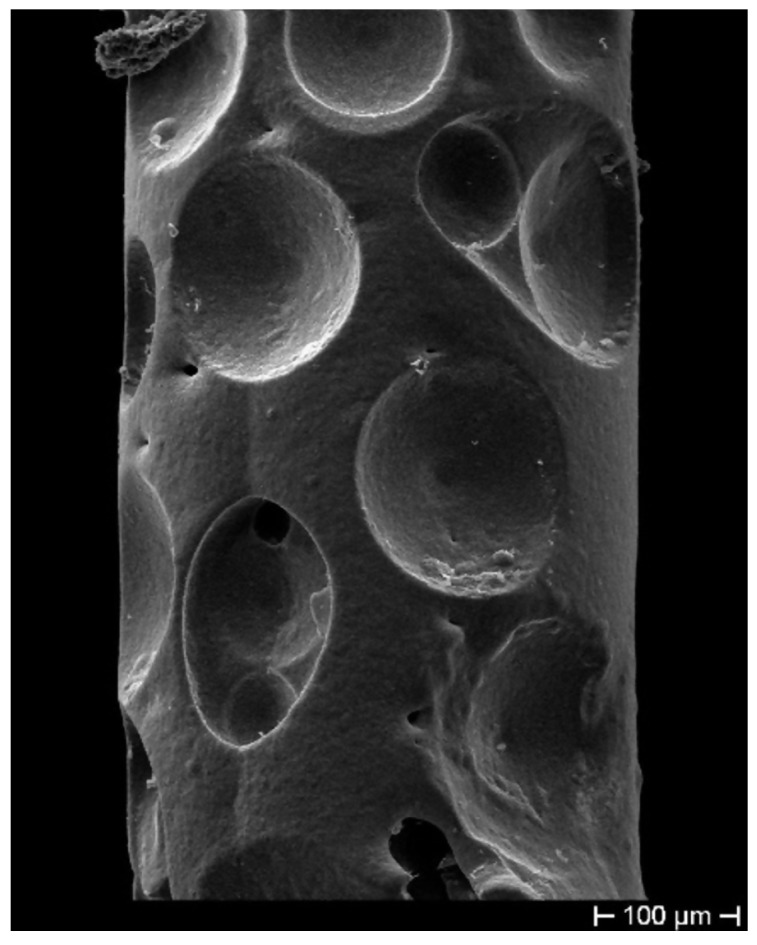
Concave formations on the coating surface as a result of hydrogen bubble presence during Ni electrodeposition (ED).

The fibers were first gilded on a stripped segment of about 50 mm in length, by means of sputtering (using an EDWARDS sputter coating, model S150B) to make the cylindrical surface electrically conductive. The gilded segment (cathode) was then subjected to ED using a custom cylindrical lead (Pb) anode submerged in the electrolyte within a cylindrical glass cell. The fiber stayed positioned along the vertical axis of the cell, slightly stretched. The controlled geometric arrangement allowed obtaining ED with uniform thickness on the cylindrical surface of the fiber. This configuration was adopted to obtain the most homogeneous radial deposition, trying in this way to avoid any anisotropy in the coating (as shown in Figure 1) and consequent non-isotropic radial stress, which can affect the grating spectrum, inducing disturbance in its behavior. Figure 1 highlights, as a consequence of a non-axial position of the cathode not perfectly aligned to the longitudinal axis of the cylindrical Pb anode (that is shown as a yellow dash-dotted line), an optical fiber with non-homogeneous thickness. The sulfate solution used as an electrolyte for the Cu ED in both coatings (bimetallic Cu–Ni layer and single metallic Cu layer) contained 25 g/L of Cu as $CuSO_{4}$ and 20 g/L of $H_{2}SO_{4}$, while the electrolyte for Ni ED contained 40 g/L of Ni as $NiSO_{4}.6H_{2}O$ and 10 g/L of $H_{3}BO_{3}$ (boric acid). The metal ED was performed for both metals at a current density of 250 A/m${}^{2}$ at RT using an Amel galvanostat, model 2053. Many samples produced during the ED trials also showed surface irregularities, as shown in Figure 2, probably due to the presence of hydrogen bubbles during Ni ED, given that Ni catalyzes the discharge of hydrogen. These hydrogen bubbles insulate the surface of the sample, preventing the deposition of more metal and generating these concave formations. Considering that the thickness is very thin, these hydrogen bubbles, although located on the surface, affect a significant volume of the coating and make it irregular, thus exacerbating the nonhomogeneous transfer of even the slightest external stress and consequently giving rise to unstable FBG spectral response.

### 2.2. Thermal Cycling

As mentioned before, the reason for coating execution is the mechanical protection of the fiber during the RP, but in turn, during such a critical thermal cycle, the metal coating is also subjected to severe thermal conditions. While during a traditional RP, the samples are exposed to HT in very long periods, and with very soft heating ramps, in this test campaign, the samples were stressed by much more critical conditions, with steeper heating rates, i.e., with the highest temperatures reached faster. To analyze the effect that the HT could have on metal coatings and that consequently could critically affect the grating spectral response, both sets and respective dummies underwent HT cycling in a tubular laboratory furnace, with temperatures ranging from RT to 800–1010 ${}^{\circ }$C for the bimetallic coating samples. Cycling from RT to 700 ${}^{\circ }$C was used for Cu-coated fibers, because of the lower melting temperature of copper. Table 1 describes which thermal cycles have been performed and on what set of samples. Dummy samples were analyzed using a scanning electron microscope (HITACHI, Tokyo, Japan, model S2500) equipped with EDS analysis.

#### 2.2.1. Recovery

While the formation of bubbles on the cathode surface was reduced by controlling and maintaining the solution pH constant, the hydrogen absorbed by Ni during ED presents two internal issues: The hydrogen absorbed during electrolysis enters the interstitial position in the Ni lattice as an atom, generating internal stress if at a high concentration (this is a first problem). Two atoms recombine to generate the molecule, and more molecules group at HT, generating porosity (internal defects) that constitutes *stress intensifier positions*. Thus, there is a need for a gentle heating (recovery, identified as first TC in Table 1) allowing hydrogen to diffuse outwards, eliminating internal stress and porosity. That recovery phase is not necessary for the Cu-coated samples, since Cu is not a hydrogen catalyst as Ni. Electrocoated FBGs underwent hydrogen recovery—carried out at 400 ${}^{\circ }$C under vacuum for 8 h—before testing the stability of the bimetallic coating during HT cycling. The recovery was therefore strictly necessary in order to reduce the large hydrogen content resulting from Ni ED. The need was highlighted by the comparison between Cu–Ni samples that underwent recovery prior to the HT cycle and samples that directly underwent the HT cycle.

**Figure 3:**
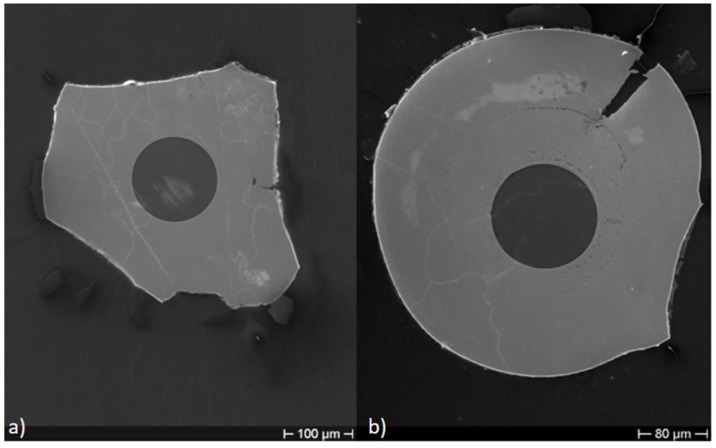
Cross-section of Cu–Ni samples with different Cu layer thickness after thermal cycling at 800 ${}^{\circ }$C. (**a**) Cu layer obtained with 90 min of ED; (**b**) Cu layer obtained with 120 min of ED.

Figure 3 shows the cross-section of Cu–Ni samples with different Cu layer thickness, after thermal cycling at 800 ${}^{\circ }$C, but without a previous recovery. Various tests were performed on the Cu–Ni samples, varying the deposition time of the Cu, until identifying the most suitable time for the subsequent deposition of Ni in 240 min. In particular, in Figure 3a, the Cu layer was obtained with 90 min of ED, while the Cu layer of Figure 3b was obtained with 120 min of ED. It is clearly shown in Figure 3a (with minor Cu thickness) that the external Ni portion is seriously compromised, while the sample of Figure 3b where the Cu thickness is greater maintains a better aspect.

**Figure 4:**
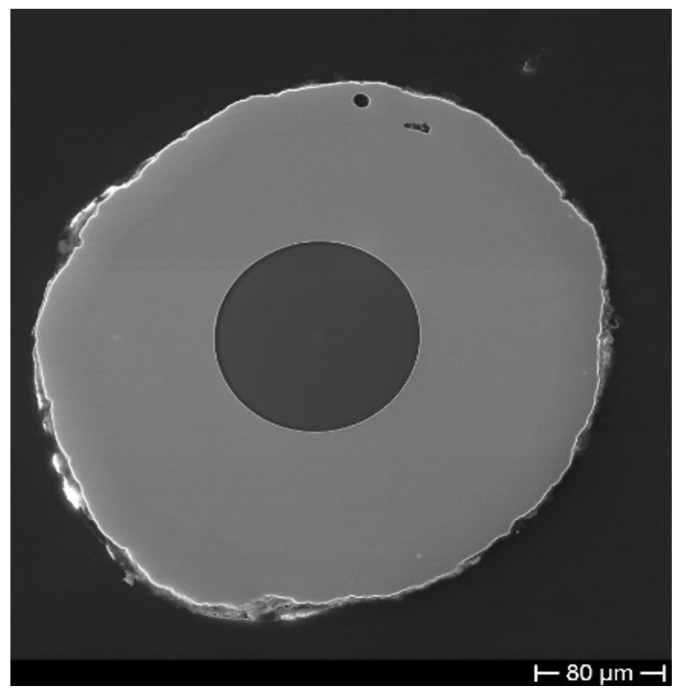
SEM micrograph of a bimetallic coating obtained by ED of Cu and Ni after thermal recovery treatment.

Instead, Figure 4 shows a Cu–Ni sample which underwent a gentle heating, increasing the T (in 8 h) from RT to 400 ${}^{\circ }$C to slowly relieve the residuals of the above-mentioned internal stresses developed during the ED. In the case of comparable thicknesses (240 min ED time) of the two metals and after the thermal recovery treatment, the fractures have quite disappeared.

**Figure 5:**
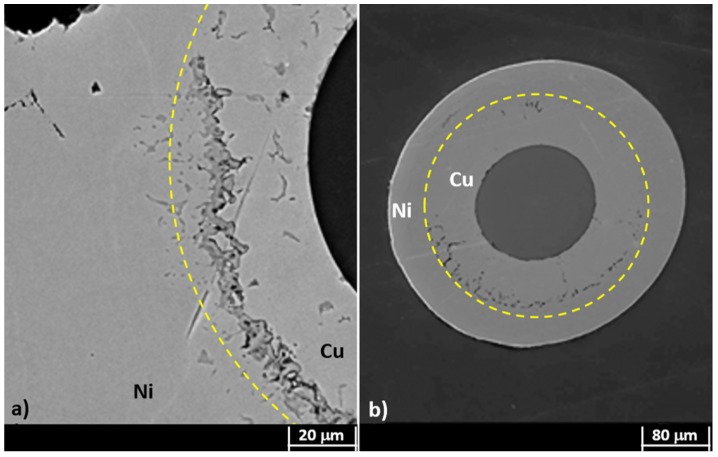
SEM micrograph of a bimetallic coating after the first heating/cooling cycle between room temperature (RT) and 800 ${}^{\circ }$C: (**a**) Cu coating thinner than the Ni one; (**b**) double layer with comparable thicknesses. Cu–Ni boundaries are highlighted by a yellow thin dotted line; it represents the region where the Cu–Ni alloy was formed.

After the recovery treatment, the samples were heated up to 800 ${}^{\circ }$C. After this first heating/cooling cycle (between RT and 800 ${}^{\circ }$C), cross-sections of dummy samples were observed at SEM, getting results as shown in Figure 5. It can be seen that the difference in thickness between the two metals affects the deposit integrity after thermal cycling treatment, although hydrogen desorption was performed. In particular, if the Cu layer is thinner than that of the Ni (Figure 5a), the slight difference in CTE of the two metals (1.65 10 ${}^{-5}$/K for Cu and 1.33 10 ${}^{-5}$/K for Ni [21]) may lead to a detachment of the outer material (Figure 5a). Nevertheless, it shall be noted that the opposite behavior can be observed if the two layers have comparable thicknesses, as shown in Figure 5b.

#### 2.2.2. Kirkendall Voids

Another observation worthy of attention in both microstructures consists in the Cu and Ni HT interdiffusion that leaves a significant presence of Kirkendall voids (KVs). The interdiffusion mechanism occurs via lattice vacancies; the formation of KVs is due to the vacancies movement in the opposite direction of the atoms. In the binary system Cu–Ni, the diffusive flux of Cu is higher than that of Ni; thus, voids form at the Cu side. The voids are due to the coalescence of the vacancies that, being in oversaturation at HT, accumulate. Even these can act as stress intensifiers and crack accelerators. Furthermore, a compressive stress is created in the Cu layer (with a greater loss of mass), while a tensile stress appears in the Ni layer, both due to the Cu/Ni interdiffusion coefficient. This irregular stress state in the coating thickness produces an adverse effect on the FBG.

**Figure 6:**
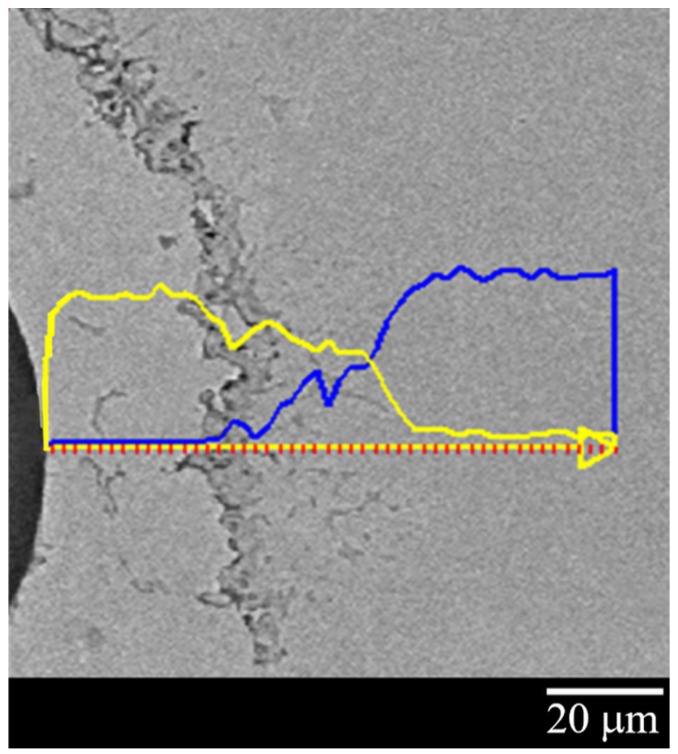
Line analysis of the Cu–Ni ED sample: Cu concentration is plotted in yellow; Ni concentration is plotted in blue.

The line analysis (Figure 6), carried out on a Cu–Ni dummy sample cross-section, shows qualitatively the Cu (yellow line) and Ni (blue line) distribution along the coating thickness and validates what was reported above. The line analysis clearly shows how, starting from the $SiO_{2}$ fiber and moving horizontally from left to right, one can find only Cu, for about the first 30 microns, until the appearance of what has been identified as the KV area (about 5–10 microns wide); then there is a Cu–Ni alloy interdiffusion zone (about 35 microns wide) with a high concentration of Cu, excluding a few microns near the only-Ni zone, that is, at the right side of the image. Figure 6 also shows that KVs are located in the Cu layer, as already demonstrated in [22,23].

Furthermore, given the system’s cylindrical geometry, the Cu–Ni interface is convex at the Cu side and concave at the Ni side. This implies that the atoms in the concave area have a greater interatomic distance than those in the convex one; therefore, the thermal fluctuations will foster the passage of atoms from the Cu convex area (compressed zone) to the Ni concave one. Thus, it has also been shown from both the micrograph and line analysis of Figure 6 that there is a greater diffusion of the Cu in the Ni with consequent formation of voids in the Cu layer. In addition, the line analysis clearly highlights the formation of a Cu–Ni alloy of variable composition between the two metal layers. According to Kirkendall’s theory, it was clear that the intrinsic diffusion rates of metals could not be described by their self-diffusion coefficient. In particular in a binary system, the intrinsic diffusion coefficient, representing the main kinetic parameter of the interdiffusion phenomenon, can be calculated by Boltzmann–Matano (B-M) analysis [24], which in turn allows the calculation of the interdiffusion coefficient.

## 3. Results

### 3.1. Coating Quality Observation

Destructive morphological analysis made on dummy samples showed that to improve the overall quality of the bimetallic coating, it was necessary to increase the thickness of both the Cu and Ni layer. That result came from cross-section analysis focused on evaluating the interlayer diffusion, which gets a key role in the stability of the bimetallic coating. In fact, two merely concentric layers can easily undergo exfoliation and pealing, and even more so when they are subject to thermal stress. In addition to concentricity, other quality parameters considered in evaluating the bimetallic coating were porosity and Kirkendal l voids. The production of samples with different layer thickness was done adopting a longer/shorter time for the ED of Cu and Ni, with equal time for both metals. The result of a better overall bimetallic coating quality with larger thickness can find explanation in the wider interface area, which can lower the effect of localized defects. As presented in the following section, the morphological analysis of the dummy samples showed that adopting an ED time of 240 min for both Cu and Ni, a potentially adequate bimetallic coating was produced. No further increase of the thickness was attempted, although in principle, it should not introduce drawbacks as it should only lead to a better compaction of the metal on the fiber, allowing a final increase of the coated sensor as for thermal sensitivity (larger thermally induced stress) [25]. That led to the production of the FBG samples from which a representative result of the spectral variation is shown in the next section.

#### Bimetallic Coating, Cu–Ni

**Figure 7:**
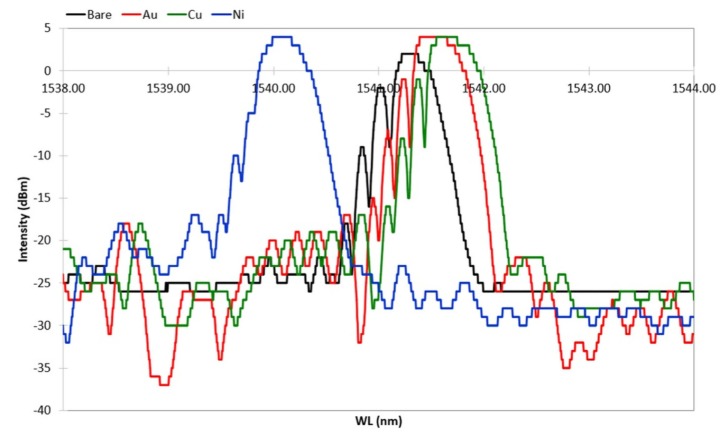
Spectral variation after the four steps of the sample production process. All plots were recorded at the same room temperature, 24 h after each production process step to allow thermal and transitory strain recovery.

The optimized ED procedure worked out with the dummy samples was adopted for the production of the FBG samples. As mentioned above, during the ED and the HT cycling, FBGs’ spectral response was continuously monitored. The grating spectral response was evaluated by comparing the spectrum fundamental parameters [26], obtained along the various steps of the ED and thermal cycles, with the spectrum parameters of the initial grating spectrum. Figure 7 shows the spectral variation along the production steps of one sample with a bimetallic (Cu–Ni) protective layer. The spectrum of the FBG after acrylate removal (first production step) is labelled *Bare*, and it is shown in black. The spectrum after gilding (second production step) is labelled *Au*, and it is shown in red. The spectrum after Cu deposition (third production step) and the spectrum after the following Ni deposition (fourth production step) are shown in green and blue, respectively. All spectra were recorded 24 h after undergoing the processing step, in order to let the sample recover from transitory stress and thermalize at the constant RT. Thus, the spectral variation shown in Figure 7 is only due to the strain applied by the step-by-step deposited coating. Figure 7 is fully representative of the spectral variation of all samples with FBG (up to the third step for a single metallic layer), which were all produced with the same setup and processing procedure as defined after the results of the preliminary production tests (dummy samples). The ED of Cu and the ED of Ni were both carried on for 240 min, as previously defined.

After gilding (in red in Figure 7), the spectrum shows a slightly larger Bragg peak value and side lobe suppression (SLS, i.e., difference between the Bragg peak and side lobe peaks). Further, a shift toward larger wavelengths can be seen, indicating that gilding produced some elongation (axial tension). Similarly, the spectral response of the FBG shows that both the peak value (power) and the noise floor increased; the impact on the signal–noise ratio SNR is ambiguous, but it was not investigated, though it may be worth future study. After Cu ED (in green in Figure 7), the spectrum suffers insignificant variations. The Bragg peak has a minor shift toward larger wavelengths, indicating that an elongation occurs. In contrast to this, after Ni ED (in blue in Figure 7), the spectrum experiences a broad shift toward lower wavelengths, maintaining the same power, whereas a large SLS suppression—present at lower wavelengths—occurs. Thus, it seems that the thickness growth affects the signal sensitivity, giving minor spectrum variation due to the background noise, improving the shape of the Bragg peak, increasing its power, and improving the sensor mechanical strength. On the whole, a large wavelength shift occurred after Ni coating (from about 1541.7 nm to 1540.1 nm), indicating that a large axial compression took place, although the spectrum shape and power of the peak remained constant. The spectral variation is dependent on the quality of the deposit, because an irregular deposit produces non-uniform radial and longitudinal stress and consequent strain. Spectra in Figure 7 show that the coating does not introduce nonhomogeneous radial or longitudinal strain on the grating (coating has good concentricity). In fact, neither birefringence (doubling of the Bragg peak) nor diffraction dispersion (broadening of the Bragg peak) occurs. The main effect is an appreciable wavelength shift with quasi-unchanged spectral shape, which can be fully explained by a homogeneous axial contraction or elongation of the Bragg grating after each step of the coating production. One more effect to be noted is a reduction of the relative amplitude of the secondary lobes at lower wavelengths, which causes a slight reduction of the full width at half maximum (FWHM) of the main peak.

### 3.2. Effect of Thermal Treatment on Spectral Variation

**Figure 8:**
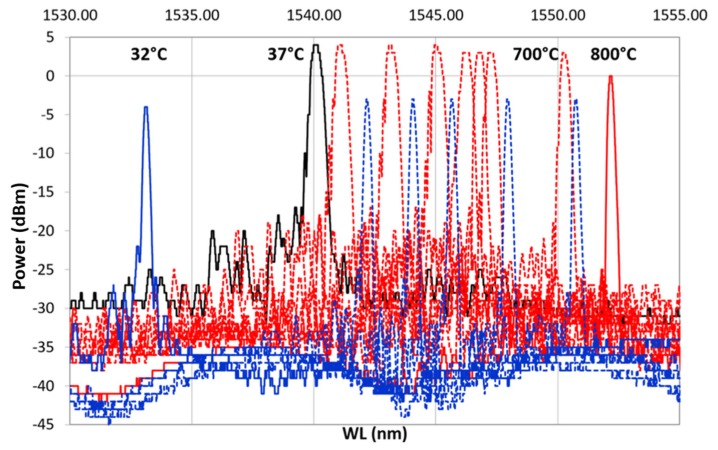
First heating/cooling cycle. In black is shown the spectrum at (RT) at the beginning of the cycle. The dotted red line represents the heating ramp from 37 ${}^{\circ }$C to 800 ${}^{\circ }$C, which is represented with a solid red line; the dotted blue line is the cooling ramp from 800 ${}^{\circ }$C to 32 ${}^{\circ }$C, shown using a solid blue line.

After ED, FBG samples underwent preliminary recovery treatment followed by thermal cycling as a final proof test (Table 1), monitoring the spectral variation. Thermal cycling was done with a heating ramp (from 37 to 800 ${}^{\circ }$C) followed by a cooling ramp (from 800 to 32 ${}^{\circ }$C). Figure 8 shows the result from one of the samples: dotted red line, spectra from the heating ramp; dotted blue line, spectra from the cooling ramp; solid black, spectrum at 37 ${}^{\circ }$C, at the beginning of the heating ramp (after the hydrogen recovery); solid red line, spectrum ad 800 ${}^{\circ }$C; solid blue line, spectrum at 32 ${}^{\circ }$C at the end of the cooling ramp. In Figure 8, labels on top of some selected spectra specify the temperature at which the spectra were recorded. As shown in Figure 8, as the temperature increases, the reference spectrum begins to shift to the right.

**Figure 9:**
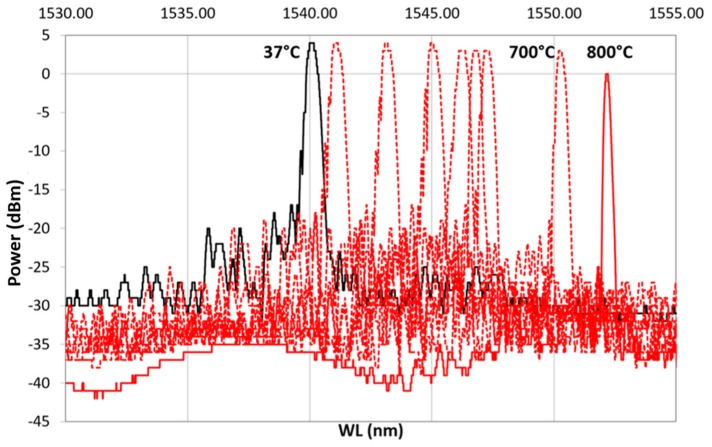
Heating ramp of the thermal cycle from 37 ${}^{\circ }$C, reference spectrum represented with a continuous black line, to 800 ${}^{\circ }$C, represented with a continuous red line.

**Figure 10:**
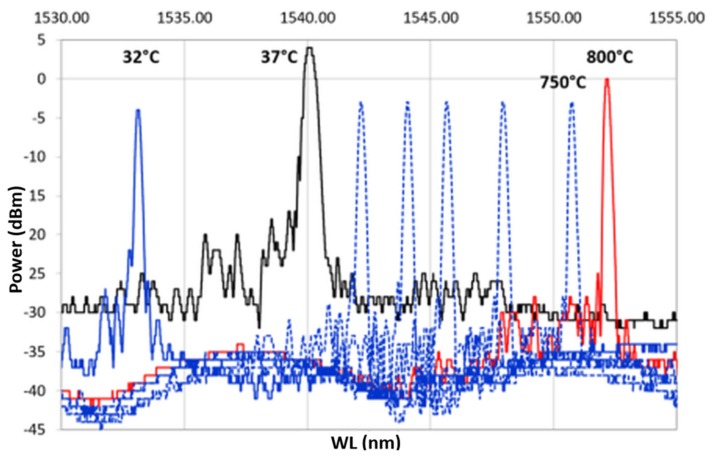
Cooling ramp of the thermal cycle from 800 ${}^{\circ }$C, represented with a continuous red line, to 32 ${}^{\circ }$C, represented with a continuous blue line.

During the heating, the spectra decreased in power, losing the side lobes, reducing in FWHM and becoming sharper. These effects became more visible between 700 and 800 ${}^{\circ }$C. This is even more evident in Figure 9, which shows only the heating ramp, and in Figure 10, which shows the cooling ramp, where the effect of temperature on the shape of the spectrum is highlighted. Thus, Figure 8, Figure 9 and Figure 10 can help to explain what happens between 700 and 800 ${}^{\circ }$C. In this temperature range, the spectrum changes because of a nonlinear stress state, probably due to the formation of the KVs. The presence of voids is undesirable in most load bearing and thermal stressed applications and would significantly undermine the integrity of coatings, as demonstrated in [22]. Figure 5, showing a micrograph of a cross-section view of the sample after the 400 ${}^{\circ }$C recovery, confirms that the diffusion phenomena are not present or at least irrelevant before this temperature, becoming significant above 700 ${}^{\circ }$C, as shown in the graphs in Figure 8, Figure 9 and Figure 10.

During the cooling phase, while the samples return to RT, the defects shown by the spectrum during heating do not recur, i.e., the spectrum shape—obtained when the maximum temperature is reached—is maintained unchanged along the cooling ramp. During cooling, the metal structure remains unchanged, and the KVs are not eliminated, since the interdiffusion phenomenon, which activates the atoms migration and ends when the temperature falls below 700 ${}^{\circ }$C, is a nonreversible process.

Furthermore, upon the sensor returning to lower temperatures, it has undergone changes such as to prevent any other spectrum variation during the new heating cycle up to 700 ${}^{\circ }$C.

This trend is better observed in Figure 8, which illustrates the complete cycle (heating and cooling), where the variation of the signal is evident in terms of power lower than at the beginning, but higher in terms of the refracted wavelength. Moreover, the difference between the initial shape at 37 ${}^{\circ }$C and the final shape at 32 ${}^{\circ }$C is remarkable. This result could justify the interdiffusion hypothesis with consequent formation of KVs and nonhomogeneous stress state along the thickness of the coating. Furthermore, the difference in wavelength between the temperature recorded at the beginning of the test (37 ${}^{\circ }$C) and the final one (32 ${}^{\circ }$C) leads to further considerations on the final conditions of the grating, which can be interpreted as the usual behavior of the grating as a result of the RP, as explained at the end of the section.

**Figure 11:**
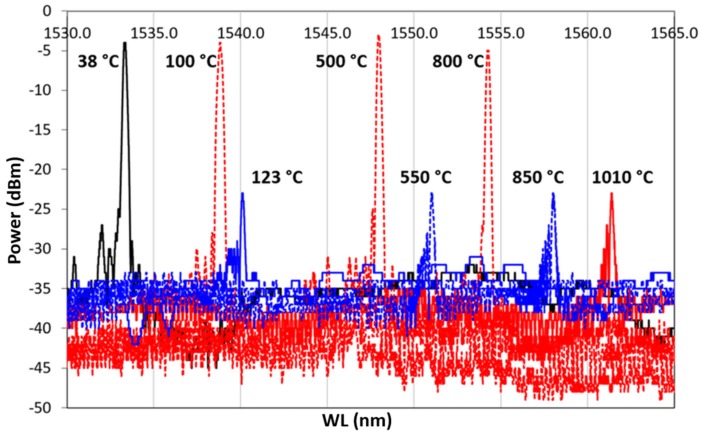
Second Heating/cooling cycle. A dotted red line represents the heating ramp, from 38 to 1010 ${}^{\circ }$C; the dotted blue line represents the cooling ramp, from 1010 to 123 ${}^{\circ }$C. A continuous line represents the reference temperature, in black the RT, that is, the initial point of the cycle, in red 1010 ${}^{\circ }$C, and in blue the last recorded spectrum, at 123 ${}^{\circ }$C.

A second heating/cooling thermal cycle was performed on the double-coated sample up to 1010 ${}^{\circ }$C and then cooled. The spectrum maintained almost the same power up to 700 ${}^{\circ }$C as in the first cycles. A further heating up to 1010 ${}^{\circ }$C reduces the power by about 70 %, as shown in Figure 11. During the cooling ramp, the test was suddenly interrupted at 123 ${}^{\circ }$C, due to the optical fiber rupture. The non-uniform tension/compression stresses, the continuous thermal shocks, and the glass embrittlement lead to optical fiber breaking at the boundary between bare fiber and metallic-coating section, where the geometry promotes a strong concentration of stress.

#### Single Metal Coating, Cu

**Figure 12:**
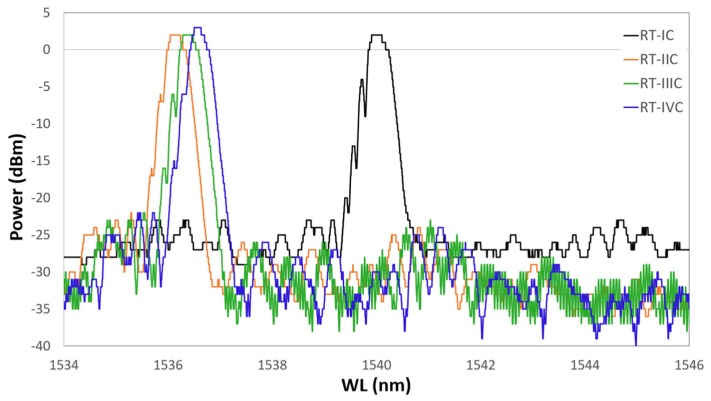
Grating spectrum at the wavelength corresponding to the RT measured at the beginning of each cycle. In black is shown the first cycle, in red the second, in green the third, and in blue the fourth.

A further hypothesis was made to explain the grating spectral response, related to the effective strength of the inner Au layer. This first micrometric layer, obtained by sputtering, is extremely delicate and is easily removable by mechanical exfoliation. This led us to suppose that, during the following metallic layer depositions and during the heat treatments, a detachment of the first layer could occur, inducing an inconsistent behavior of the grating. This question has been dispelled by considering that a possible detachment of the first layer would have led to an almost total separation between the surface of the optical fiber and the metallic coating, generating a separation interface (an interspace) between them, leading to a further inconsistent behavior of the spectrum. Thus, a test was carried out with an optical fiber sample coated with Au and Cu, both deposited as described above. The sample underwent four heating/cooling thermal cycles, from RT (20 ${}^{\circ }$C) to 700 ${}^{\circ }$C. The heating ramp lasted about three hours, while the cooling was performed by turning off the heat source and letting the system cool down during the night (about 12 h). The RT spectrum was recorded before starting each thermal cycle. Figure 12 shows the spectrum of the same grating at the wavelength corresponding to the RT measured at the beginning of each cycle.

**Figure 13:**
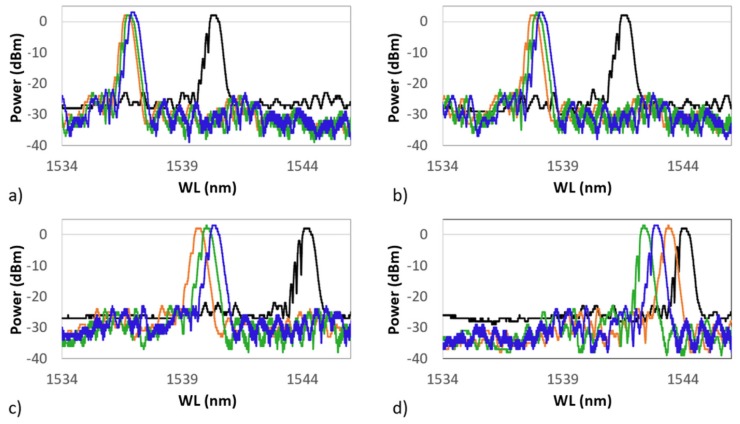
Grating spectra grouped by temperature value. (**a**) The grating spectrum at 100 ${}^{\circ }$C; (**b**) at 300 ${}^{\circ }$C; (**c**) at 500 ${}^{\circ }$C; and (**d**) at 700 ${}^{\circ }$C. After the first cycle, all following cycles begin around the new wavelength value at RT. In black is shown the first cycle, in red the second, in green the third, and in blue the fourth.

The black plot represents the grating spectrum as it was 24 h after the Cu coating, and it is defined as the first heating cycle. For each spectrum, the sample is recorded while at RT, just before starting the heating which brings it up to 700 ${}^{\circ }$C. In red is shown the RT spectrum at the beginning of the second cycle, in green the spectrum at the beginning of the third cycle, and finally, in blue the spectrum shown at the beginning of the fourth cycle. During tests, the RT value was around 21 ± 1 ${}^{\circ }$C for each cycle. The four frames of Figure 13 show four spectra of the same grating grouped by temperature value. In each frame, the grating is kept at a constant temperature. In (a) is shown the grating spectrum at 100 ${}^{\circ }$C, in (b) at 300 ${}^{\circ }$C, in (c) at 500 ${}^{\circ }$C, and in (d) at 700 ${}^{\circ }$C, during four succeeding thermal cycles. In each frame, each cycle is represented as it is in Figure 12. The latter suggests that a very fast annealing (first thermal cycle) destabilizes the grating, inducing a sort of regeneration which involves the shifting of the central wavelength value towards the left. In the following cycles, the grating reaches, unless showing small differences, a well-defined wavelength for each of the temperatures set in the four frames of Figure 13.

The four graphs of Figure 13 show how, following the first thermal cycle (always shown in black in Figure 12 and Figure 13), the grating maintains a more coherent behavior in the three following cycles. At 100 ${}^{\circ }$C (Figure 13a) , 300 ${}^{\circ }$C (Figure 13b), and 500 ${}^{\circ }$C (Figure 13c), the spectrum moves proportionally to the right (increasing wavelength) as a result of the heating. At 700 ${}^{\circ }$C (Figure 13d), the sensor spectra tend to group themselves around the same wavelength (around 1543.00 nm). This effect could be ascribed to the strongly nonlinear behavior of the photoelastic coefficient at HT. This test demonstrates that in the same range of temperatures, and in the absence of the second metal, the anomalies detected in the presence of the second metal (as in the case of the Ni–Cu bimetallic coating) are not registered and that there are no detachments at the interface between optical fiber and metal coating. This would also confirm the behavior shown in Figure 8 and Figure 10, where the last recorded WL (at 32 ${}^{\circ }$C) was moved much further to the left than the RT value preceding the thermal cycle (at 37 ${}^{\circ }$C).

## 4. Conclusions

This work presents some results of a wider research on metallic coatings for FBGs that our team has been conducting for several years, on applications of FBG in a harsh environment, especially at extreme temperatures (from cryogenics to HT). These gratings need to undergo critical thermal treatments; therefore, there is a need for a better-performing mechanical protection. For that reason, a double-layer Cu–Ni coating, on gratings specifically written for RP purposes, was investigated. Cu allows a better adhesion to the first very thin Au substrate and permits easily obtaining a continuous deposit, while Ni protects the system from higher temperatures. Moreover, the two metals, at HT, interdiffuse, generating cupronickel alloys. These present both mechanical strength and resistance to aggressive environments greater than those of the two elements separately. The coating, following a preliminary recovery cycle, performed at 400 ${}^{\circ }$C to release the hydrogen absorbed by the Ni layer, undergoes a very critical (fast) RP. Up to 700 ${}^{\circ }$C, KV development does not occur, and the grating maintains a consistent behavior. After 700 ${}^{\circ }$C, KVs appear and increase with the increasing temperature, leading to a clearly visible grating instability in the range of 700–800 ${}^{\circ }$C. Thermal cycling at HT (800–1010 ${}^{\circ }$C) emphasized two critical issues: First, the formation of KVs in the Cu zone of the Cu–Ni deposit, in addition to being a factor of stress concentration, can produce a sensor malfunction as a consequence of the creation of an irregular stress state. Second, the spectrum peak power is reduced though the sensor maintains a correct operation up to 800 ${}^{\circ }$C. Over 800 ${}^{\circ }$C and particularly when the cycle reaches 1010 ${}^{\circ }$C, the power drops to 30%, and that value is maintained during cooling.

Regardless of the thermo-optical coefficient effects (the FBG’s thermal sensitivity depends on it), the paper focused on the interface between Cu and Ni where, over 700 ${}^{\circ }$C, the reciprocal displacement of the two layers and the consequent opposite tensions generate a stress-concentration *belt*. The test carried out with the Cu-coated samples showed that, in the case of a single metal, this state of internal tension does not occur, since the KVs cannot be generated. Moreover, it confirmed that the spectrum instability shown is not due to a coating detachment, as it continues operating for several (four) successive thermal cycles. Although the bimetallic coating would allow the use of the FBG up to 400 ${}^{\circ }$C, and for short periods up to 700 ${}^{\circ }$C, the regeneration following such a rapid process cannot be considered complete. The critical issues attributable to the deposition of the double layer could be overcome by optimizing the metals deposition time, considering that, the greater the interface surface, the better the adhesion between the two layers. Moreover, a larger exchange surface encourages the interdiffusion between the layers and a larger radius (as the radius increases, i.e., the deposit thickness, the surface curvature decreases) reduces the stress concentration. A further contribution to eliminate the problems encountered during the tests could be to perform a less steep heating ramp (i.e., a more gradual increase in temperatures, over longer times), as this could ease the interdiffusion, allowing it to take place in a uniform manner without generating discontinuities in the internal structure of the coating. However, with increasing thermal cycle time, the Kirkendall effect may play a more important role in the formation of voids. The longer the HT exposition interval is, the bigger the number of voids and the larger the diameter of voids will be [27]. We must therefore seek a compromise, that is, a long enough interval to allow grating stabilization but not so long as to allow the formation and growth of KVs. In addition to the above, this works highlights the importance of performing a recovery treatment for the hydrogen desorption from Ni that constitutes an additional embrittlement factor of the bimetallic deposit.

## Figures and Tables

**Table 1:** Thermal cycles’ (TC) resume.

Thermal	Coating	T (${}^{\circ }$C)	Duration	Purpose	Effect
Test	Type				on FBG
Recovery	CuNi	RT-400	8 h	Eliminate internal stresses.	No issues arisen
	Samples			Hydrogen desorption	
I TC	CuNi			Evaluation of HT	Kirkendall voids.
Heating	Samples	37–800	3 h	effect on coating	Spectrum power
Cooling		800–32	12 h		decay at 800 ${}^{\circ }$C
II TC	CuNi			Evaluation of HT	Further spectrum
Heating	Samples	32–1010	3 h	effect on coating	power decay
Cooling		1010–123	12 h		Sample break
I to IV TCs	Cu			Comparison with	
Heating	Samples	20–700	3 h	CuNi samples	
Cooling		700–20	12 h		

## Abbreviations

The following abbreviations are used in this manuscript:

CTE	Coefficients of thermal expansion
CVD	Chemical vapor deposition
ED	Electrodeposition
FBG	Fiber Bragg grating
FWHM	Full width at half maximum
HT	High temperatures
KV	Kirkendall void
PVD	Physical vapor deposition
RP	Regeneration procedure
RT	Room temperature
SEM	Scanning electron microscope
SLS	Side lobe suppression
SNR	Signal–noise ratio
TC	Thermal cycle
UV	Ultraviolet
